# Sonodynamic Effects of a Novel Ether-Group Modified Porphyrin Derivative Combined With Pulsed Low-Intensity Ultrasound on PC-9 Cells

**DOI:** 10.3389/fphar.2021.792360

**Published:** 2021-12-06

**Authors:** Yinghua Jin, Qi Zhou, Jianxiong Geng, Qingwei Meng, Zixin Wei, Meijuan Ding, Jing Zhou, Yuan Zeng, Wenwu Cao, Fang Liu, Yan Yu

**Affiliations:** ^1^ Department of Medical Oncology, Harbin Medical University Cancer Hospital, Harbin, China; ^2^ Department of Instrument Science and Engineering, School of Electronic Information and Electrical Engineering, Shanghai Jiao Tong University, Shang Hai, China; ^3^ Department of Mathematics, The Materials Research Institute, Pennsylvania State University, University Park, PA, United States; ^4^ Condensed Matter Science and Technology Institute and School of Instrumentation Science and Engineering, Harbin Institute of Technology, Harbin, China

**Keywords:** porphyrin derivative, sonodynamic therapy, pulsed low-intensity ultrasound, lung cancer, sonosensitizer

## Abstract

Sonodynamic therapy (SDT) is a developing modality for cancer treatment based on the synergistic effect of ultrasound and chemical compounds which are known as sonosensitizers. The development of more efficient sonosensitizers has become an urgent issue in this field. In this study, a novel porphyrin derivative (BBTPP) mediated SDT was evaluated on PC-9 cells. Pulsed low-intensity ultrasound (PLIU) was used for its little thermal and mechanical damage. The accumulation of drugs in cells was evaluated through porphyrin fluorescence, and the cytotoxicity of BBTPP was evaluated using a cell counting kit-8 assay. The sonodynamic effect was investigated by Hoechst 33342/PI and Annexin V-FITC/PI double staining, which showed an apoptotic rate of 18.87% in the BBTPP-SDT group, as compared with 1.71%, 1.4%, 1.57%, 3.61%, 11.18% in the control, BBTPP, hematoporphyrin monomethyl ether (HMME), ultrasound, and HMME-SDT groups, respectively. The sono-toxic effect of BBTPP was significantly superior to HMME. Our results showed that BBTPP-SDT resulted in much higher intracellular reactive oxygen species (ROS) and lipid peroxidation levels which were evaluated by 2′,7′-dichlorodihydrofluorescein diacetate (H_2_DCFDA) and Liperfluo assay, respectively. The expressions of Bax, Bcl-2, caspase-9, caspase-8, and cleaved caspase-3 proteins were evaluated to investigate the apoptotic mechanism of BBTPP-SDT. The results of this study showed that the combination of BBTPP and PLIU induced the generation of ROS, resulting in lipid peroxidation, and activated both the extrinsic and intrinsic apoptotic pathways of PC-9 cells. Our results also suggested that the ether group introduced in the side chain of porphyrin could enhance the sono-toxicity of porphyrin-based sensitizers under the sonication of PLIU. These results supported the possibility of BBTPP as a promising sonosensitizer, and an appropriate side chain could enhance the sono-sensitivity of porphyrins.

## Introduction

Sonodynamic therapy (SDT) involves the use of sonosensitizers that only become cytotoxic upon exposure to ultrasound (US) (Yumita et al., 1989; Dolmans et al., 2003; Rengeng et al., 2017). SDT has displayed impressive anticancer effects (Rosenthal et al., 2004; Trendowski, 2014; Wood and Sehgal, 2015; Wan et al., 2016) and inhibition of atherosclerotic plaque progression (Sun et al., 2019) in various studies. Capitalizing on the deep penetration into biological tissues noninvasively of US, the superiority of SDT in treatment emerges from the ability to locally activate preloaded sonosensitizers, which have little toxicity to normal tissues, on lesions buried deeply in tissues.

Pulsed low-intensity ultrasound (PLIU), with an acoustic intensity under 3.0 W/cm^2^, has minimum thermal and mechanical effects on human tissues. Around the frequency of 1 megahertz, low-intensity US is capable of generating acoustic cavitation and therefore apparent sonochemical activities (Brotchie et al., 2009), which makes it recommended to be employed in SDT (Jiang et al., 2019). With appropriate sonication apparatus, a potent sonosensitizer is indispensable for the optimization of SDT efficacy. As the most concerned type of sensitizer, porphyrin and its derivatives valid their ability for selective accumulation inside disease cells and little toxicity when applied to both SDT and photodynamic therapy (PDT) (Chen et al., 2014). Numerous novel porphyrin-related sonosensitizers (Kuroki et al., 2007; Tsuru et al., 2012), including metal-based porphyrin (Giuntini et al., 2018) and porphyrin-based nanoparticles (Qian et al., 2016; Canavese et al., 2018) have been synthesized and proven with enhanced cell toxicity under US.

Diverse forms of cytotoxic mechanisms that predominately contribute to the cytotoxicity of porphyrin-mediated SDT have been reported (McHale et al., 2016; Choi et al., 2020). Generating excessive amounts of reactive oxygen species (ROS) that could exert lethal oxidative damage to cellular organs has been considered the most effective way to achieve proper cytotoxicity (Wan et al., 2016). Although ROS always plays a harmful role, moderate levels of ROS are pivotal for normal cellular life (Holmström and Finkel, 2014). Even if certain levels of ROS could promote tumor progression, the tumor cells are more fragile when facing oxidative disorder. When the oxidative balance tilts to excessive levels of ROS, it can still be detrimental to cancer cells (Perillo et al., 2020). Cell membranes and membranous organelles such as mitochondria are particularly vulnerable to ROS due to lipid peroxidation, which could end up with cell apoptosis (Kiang et al., 2012).

Massive efforts have been made to elucidate the relationship between the structure of sonosensitizer and its sonosensitivity and to discover more effective sonosensitizers. It has been reported that the sono-cytotoxicity of metal porphyrin derivatives varied according to the metal moiety in the macrocycle. It seems that the effectiveness of sonosensitizers was closely related to the photosensitivity of the porphyrin macrocycle’s electronic structure to sonoluminescence. However, the light intensity of sonoluminescence induced by the low-intensity US or the therapeutic US is serval orders lower than lasers in PDT applications (Beguin et al., 2019). As a result, this strategy could hardly be valid for PLIU based SDT applications. On the other hand, there were speculations that the outcome might depend on the side-chain structure rather than the macrocycle (Tsuru et al., 2012). The synergistic effect of US and drugs on cells was greatly influenced by the amphiphilicity of the side chain molecules (Dougherty, 1987; Miyoshi et al., 2003; Kale et al., 2017). Several results showed that the cytotoxicity of agents that are devoid of photosensitivity could still be enhanced by US (Hachimine et al., 2007).

With this in mind, we suppose that the tumor cell-killing efficacy of SDT could be enhanced by improving the sonochemical reactivity of the side chains of porphyrin sonosensitizers. In this study, we have introduced and tested a potent novel sonosensitizer (a porphyrin derivative)-BBTPP [butyl 3-(3-(3-(2-(2-hydroxyethoxy)ethoxy)-3-oxopropyl)-12,17-bis(1-(2-(2-hydroxyethoxy)ethoxy)ethyl)-2,8,13,18-tetramethylporphyrin-7-yl)propanoate]. In the side-chain structure of BBTPP, we have introduced multiple ether groups. The ether groups could be easily oxidized due to elevated temperature conditions that shall be formed during ultrasonic cavitation and generate peroxyl radicals. The cell-killing efficacy of BBTPP under the irradiation of pulsed low-intensity focused ultrasound (PLIFU) was evaluated and compared with hematoporphyrin monomethyl ether (HMME), which had a similar porphyrin ring structure but different side chains. Possible mechanisms of BBTPP-mediated SDT were also evaluated.

## Materials and Methods

### BBTPP and HMME

A novel porphyrin derivative designated BBTPP [butyl 3-(3-(3-(2-(2-hydroxyethoxy)ethoxy)-3-oxopropyl)-12,17-bis(1-(2-(2-hydroxyethoxy)ethoxy)ethyl)-2,8,13,18-tetramethylporphyrin-7-yl)propanoate] was design and synthesized by Harbin Institute of Technology. HMME was purchased from Shanghai Xianhui Pharmaceutical Co. Ltd.

### Cell Line

Human lung cancer cell lines PC-9 was obtained from the Institute of Cancer Prevention and Treatment, Harbin Medical University, and maintained in RPMI 1640 (Gibco) supplemented with 10% fetal bovine serum (Gibco) and 1% penicillin/streptomycin antibiotics (Beyotime). Cultures were incubated in a humidified atmosphere of 95% air and 5% carbon dioxide (CO_2_) at 37 °C.

### Detection of Drug Fluorescence

Cells were seeded at a density of 10^5^ cells per dish in a 35 mm cell culture dish (Corning) overnight. The culture medium was replaced with a fresh culture medium with BBTPP. Cells were imaged using the EVOS FL Auto Cell Imaging System (Thermo Fisher Scientific) after 2 h incubation.

### BBTPP Cytotoxicity Assay

Cytotoxicity was evaluated with a Cell Counting Kit-8 (CCK-8; Dojindo). BBTPP powder was dissolved to 10 mg/ml in dimethyl sulfoxide (DMSO; Sigma) and further diluted into the desired concentration. Cells were seeded at a density of 10^4^ cells/well in a 96-well plate. After 24 h, cells were incubated with different doses (64, 32, 16, 8, 4, and 2 μg/ml) of BBTPP for another 24 h. Then, the culture medium was replaced with a fresh culture medium and incubated with CCK-8 solution for 3 h. The absorbance was read at 450 nm using an ELx808 microplate reader (Bio-Tek Instruments). Relative cytotoxicity was expressed as a percentage of controls.

### Pulsed Low-Intensity Focused Ultrasound (PLIFU) Treatment

US waves were produced by an 858 kHz single element focused ultrasonic transducer containing a 50 mm-diameter spherical cap-shaped piezoelectric ceramic. The transducer worked at tone burst mode and was driven by an RF power amplifier (1020L, Electronics and Innovation). The amplifier was fed by a signal generator (DG4062, Rigol) which controlled the ultrasonic treatment parameters, including the frequency, amplitude, duty cycle, pulse repetition period and treatment time. The transducer was submerged in a tank of degassed water which was prepared by vacuum degassing before each experiment. A calibrated needle hydrophone (HNC-1000, Onda) carried on a 3-axis precise translation stages (KA-400, Zolix) was used to measure the free-field acoustic pressure distribution around the focus region. The acoustic pressure was calculated from the peak-to-peak amplitude of voltage on the hydrophone in one pulse repetition period. The focal length is 34.8 mm according to our test results. The focal region has an ellipsoid shape with 5.1 mm in the axial direction and 2.2 mm in the lateral direction.

Tumor cells suspensions were treated with PLIFU in a polystyrene cuvette (Length/Width/Height = 10 mm/10 mm/45 mm) at room temperature for 5 min. The cuvette wall had been ground to a thickness less than 0.1 mm for minimizing the US wave reflection. 700 μL cell suspension without any visible bubbles was used for each treatment. The transducer was horizontally oriented and aimed at the center of cell suspensions. Both the transducer and the cuvette were mounted on a special-designed monolithic fixture to avoid any possible alignment issues that might decrease the sonication consistency. An anechoic rubber plate (HAMA, Precision Acoustics) was placed at the end of the US beam to absorb the transmitted US waves. In this study, the spatial maximum peak negative pressure on the focal spot was 0.39 MPa (2.5 W/cm^2^). The duty cycle and pulse repetition period are 20% and 20 m, respectively. The obvious motion of cells in liquid could be observed when US was applied which mixed the suspension during treatment. The focus region of US is approximately 3 mm below the liquid surface. Neither acoustic fountain nor shape change on the liquid surface was observed during sonication. The liquid temperature variation at the transducer focus was less than 0.2°C during sonication (measured by a needle-type thermal couple in preliminary experiments), which rules out thermal injuries to the cells. In ROS and lipid peroxidation assays, cell culture dishes planted with tumor cells were used instead of the cuvette. The cell culture dish was sealed by a 20 μm polyethylene film and exposed to PLIFU perpendicularly for 5 min through the film.

### Detection of Apoptosis

Apoptosis was evaluated through morphology (double staining with Hoechst 33342/PI; Solarbio) and analysis of phosphatidylserine externalization (Annexin V-FITC/PI double staining, BD Biosciences).

Morphological changes of apoptosis and necrosis in PC-9 cells treated with SDT were demonstrated by fluorescence microscopy. PC-9 cells were seeded in a complete medium on 35 mm cell culture dishes (Corning) at a density of 10^5^ cells per well for 24 h. Then, the cell medium was replaced with a fresh complete medium with or without BBTPP (concentration of 4 μg/ml)/HMME (concentration of 8 μg/ml) to further incubate for 2 h before US treatment. PC-9 cells exhibit higher cellular uptake of BBTPP than HMME, so different doses of agents were used in this study to obtain equivalent intracellular concentrations. The concentration of BBTPP and HMME were normalized by detection of intracellular fluorescence of PC-9 cells after incubation with different chemicals (see supplementary materials). After treatment, cells were washed with cold phosphate-buffered saline (PBS) 3 times and stained with Hoechst 33342-PI according to the manufacturer’s instructions. Cell images were performed using the EVOS FL Auto Cell Imaging System (Thermo Fisher Scientific).

Analysis of phosphatidylserine externalization was examined by flow cytometry using Annexin V-FITC/PI. Cells were cultured in a 25 cm^2^ culture flask (Corning) for 24 h and then replace medium with a fresh complete medium with or without BBTPP/HMME for 2 h. Before US treatment, cells were trypsinized and resuspended into a polystyrene cuvette. After sonication, cells were stained with a combination of Annexin V-FITC/PI according to the manufacturer’s protocol. Final fluorescence was obtained in 10,000 events on flow cytometry (Cytomics FC500; Beckman Coulter) and analyzed using FlowJo software (Version 10.7.2.).

### ROS Detection

The ROS-reactive fluorescent probe 2′,7′-dichlorodihydrofluorescein diacetate (H_2_DCFDA, Sigma) is used as an indicator for ROS. PC-9 cells were seeded into 35 mm cell culture dishes (Corning) at a density of 10^5^ cells/well for 24 h. According to the manufacturer’s protocol, the H_2_DCFDA at a final concentration of 10 μM in RPMI-1640 medium (serum-free) was added and incubated for 30 min in the dark, in a conventional incubator (37°C, 5% CO_2_). Wash twice with PBS, add fresh medium containing BBTPP (4 μg/ml, only fresh medium in control and sonication groups), and incubate for 2 h before sonication. After sonication, wash the cells with PBS twice and image with the EVOS FL Auto Cell Imaging System and quantified using ImageJ software.

### Detection of Lipid Peroxidation

The Liperfluo (Dojindo) was used to detect lipid peroxidation according to the manufacturer’s protocol. PC-9 cells (1×10^5^) were seeded on tissue culture dishes (Corning, 35 mm × 10 mm). Twenty-4 hours later cells were treated with fresh complete medium with or without BBTPP (concentration of 4 μg/ml). After 2 h drug incubation, cells were washed with PBS 2 times and replaced with RPMI-1640 medium (serum-free) supplemented with 1 µM Liperfluo reagent. Before sonication, cells were washed with RPMI-1640 medium (serum-free) twice. After sonication, cells were washed with PBS twice and image with the EVOS FL Auto Cell Imaging System and quantified using ImageJ software.

### Protein Extraction and Western Blotting

After treatment, cells were trypsinized, washed twice in ice-cold PBS, and lysed using RIPA (Solarbio) buffer containing protease inhibitor phenylmethylsulfonyl fluoride (PMSF, Solarbio). Sonicated cell lysates were centrifuged at 12,000 rpm for 15 min at 4°C. After loading equal amounts, proteins were separated using SDS-PAGE gels and transferred to nitrocellulose membranes (Pall Corporation). The membranes were blocked with 5% skim milk (Difco™ Skim Milk; BD Biosciences) in PBS for 1 h at room temperature and incubated with primary antibodies against caspase-9 (Proteintech, 66,169-1-lg, dilution 1:1,000), caspase-8 (Proteintech, 66,093-1-l g, dilution 1:5000), Bax (Proteintech, 60,267-1-l g, dilution 1:10000), Bcl-2 (Proteintech, 60,178-1-l g, dilution 1:5000), cleaved caspase-3 (Cell Signaling Technology, #9661, dilution 1:1,000), *β*-actin (ZSGB-BIO, TA-09, dilution 1:1000) overnight at 4°C. After incubating with primary antibodies, membranes were washed with TBS-T (Beyotime), and were incubated for 1 h with their respective secondary antibodies, including DyLight 800-labeled goat anti-mouse IgG (5230-0402, dilution 1:5000, KPL) and DyLight 680-labeled goat anti-rabbit IgG (5230-0415, dilution 1:5000, KPL). After this incubation, the membranes were washed with TBS-T, and bands were visualized on an ODYSSEY ® CLx scanner (LI-COR Biosciences) using both 700 and 800 nm wavelength channels and quantified using ImageJ software.

### Statistical Analysis

Experiments were repeated three times. All values are expressed as mean ± standard deviation. The difference between groups was determined using a one-way analysis of variance followed by Fisher test. *p* < 0.05 was considered a statistically significant difference.

## Results

### The Structure, Fluorescence, and Toxicity of BBTPP

**FIGURE 1:**
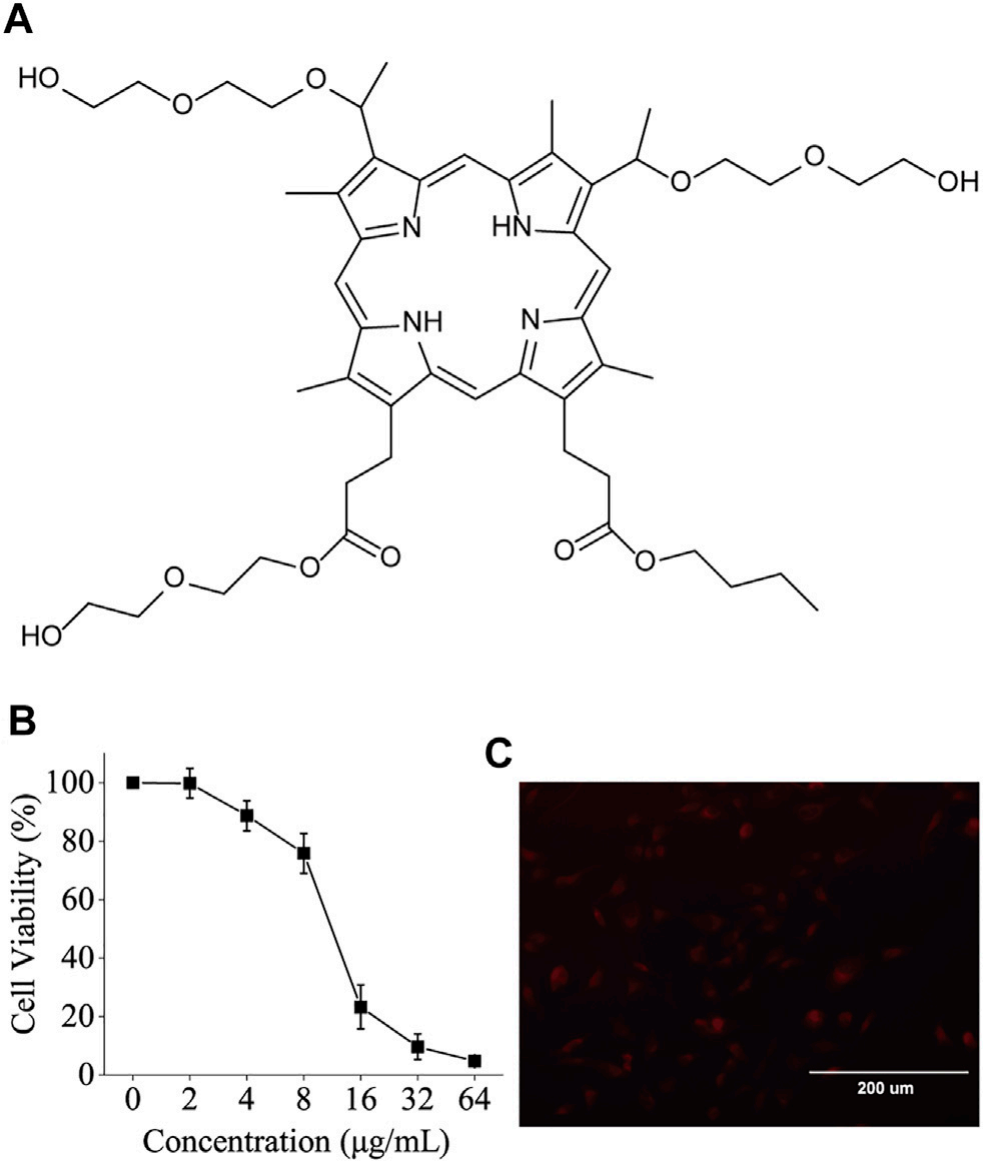
**(A)** Chemical structure of BBTPP **(B)** Dependence of BBTPP concentration on the cell viability of PC-9 cells 24 h post-administration **(C)** Fluorescence image of 4 μM BBTPP on PC-9 cells 2 h post-administration, indicating intracellular accumulation of BBTPP.

The structure of BBTPP is illustrated in Figure 1A. To choose a suitable concentration for further experiments, the cytotoxic effect of BBTPP was evaluated on PC-9 cells through cell viability. Though the incubation time of experiments was 2 h, the time for cytotoxicity was set 24 h to assess the long-time effect. After incubation of various concentrations of chemicals for 24 h, the results of CCK-8 assays showed a concentration-dependent toxic effect on PC-9 cells. As shown in Figure 1B, no obvious cytotoxicity could be found when the concentration was lower than 4 μg/ml. Thus, we employed a concentration of 4 μg/ml in the following exploration.

Metal-free bases of porphyrins could exhibit a red fluorescence in the range from 600 to 730 nm (Quiroz-Segoviano et al., 2014). The fluorescence microscopic images shown in Figure 1C indicates that BBTPP could efficiently enter PC-9 cells as evidenced by the presence of strong intracellular red fluorescence after 2 h incubation of BBTPP.

### Sonosensitivity Detection of BBTPP

**FIGURE 2:**
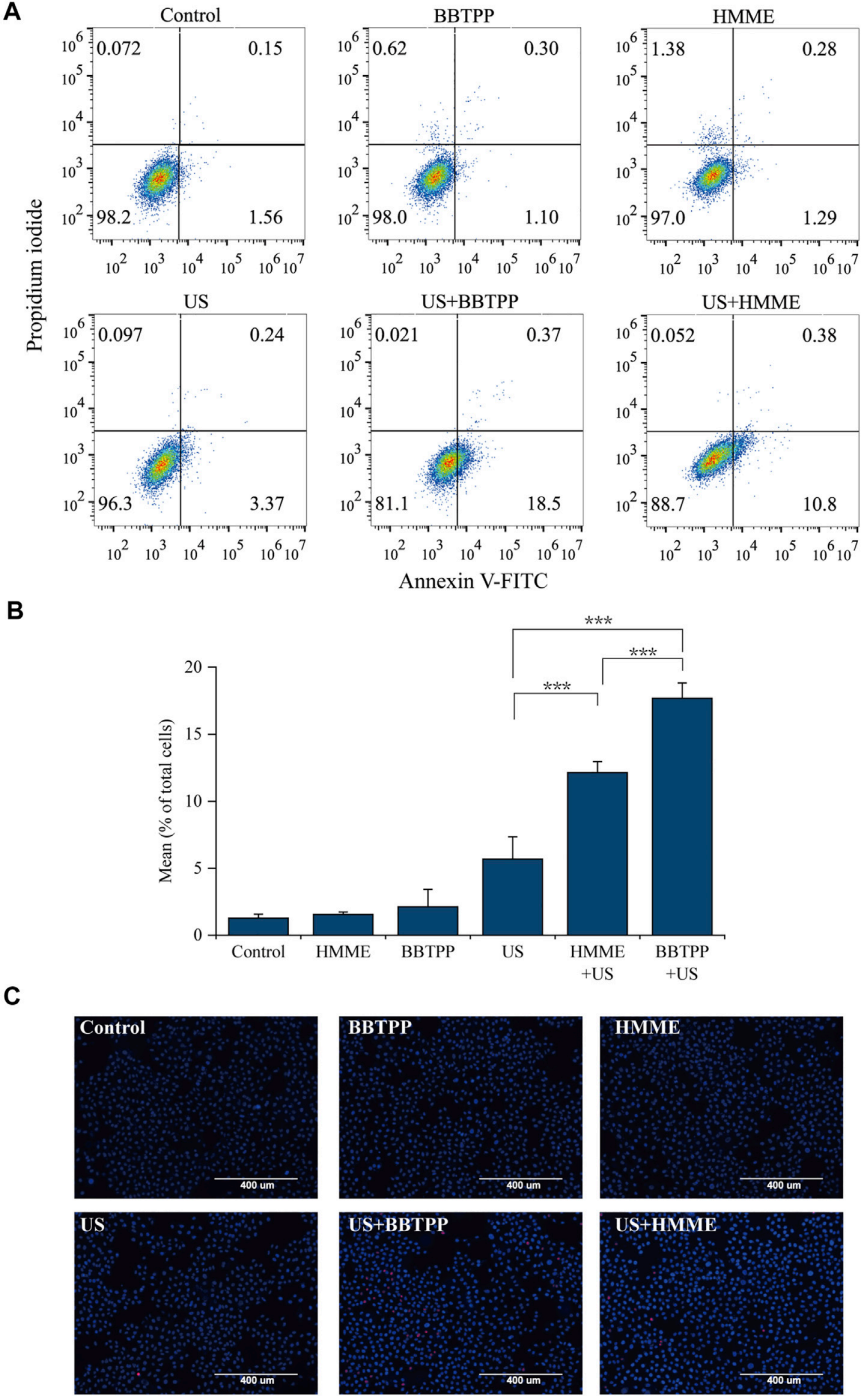
Effects of BBTPP and HMME induced apoptosis under sonication were determined with Annexin V-FITC/PI and Hoechst 33342/PI double-staining assays in PC-9 cells **(A)** Flow cytometry results with Annexin V-FITC/PI double-labeling in PC-9 cells **(B)** A statistical graph of the ratio of apoptosis among different experiment groups in Annexin V-FITC/PI staining. The data were presented as the mean ± standard deviation. The apoptotic cells included the Annexin V+/PI− cells and Annexin V+/PI + cells **(C)** Fluorescent assay of Hoechst 33342/PI staining. US: ultrasound; ***p<0.001.

To detect the sonosensitivity and efficacy of BBTPP, cell death (apoptosis/necrosis) was evaluated with Annexin V, Hoechst 33,342, and PI after SDT. Cells were equally divided into six groups (control, BBTPP, HMME, US, BBTPP-SDT, and HMME-SDT). Quantitative analysis of apoptosis was analyzed by flow cytometry (using AnnexinV/PI). The total of early (Annexin V+/PI−) and late (Annexin V+/PI+) apoptotic rate of BBTPP-SDT group was 18.87%, as compared with 1.71%, 1.4%, 1.57%, 3.61%, 11.18% in the control, BBTPP, HMME, US, and HMME-SDT groups respectively (Figures 2A,B). Under fluorescence microscopy, a considerable proportion of cells in the SDT group displayed characteristics of apoptosis with condensed and fragmented nuclei after being stained with Hoechst 33342/PI (Figure 2C). Dead cells were stained with PI and appeared as a red color. These results demonstrated that BBTPP could induce cell damage under our sonication conditions that did not induce cell damage alone. In addition, the efficacy of BBTPP was superior to HMME under the same US apparatus in this study.

### Production of Reactive Oxygen Species and Lipid Peroxidation

**FIGURE 3:**
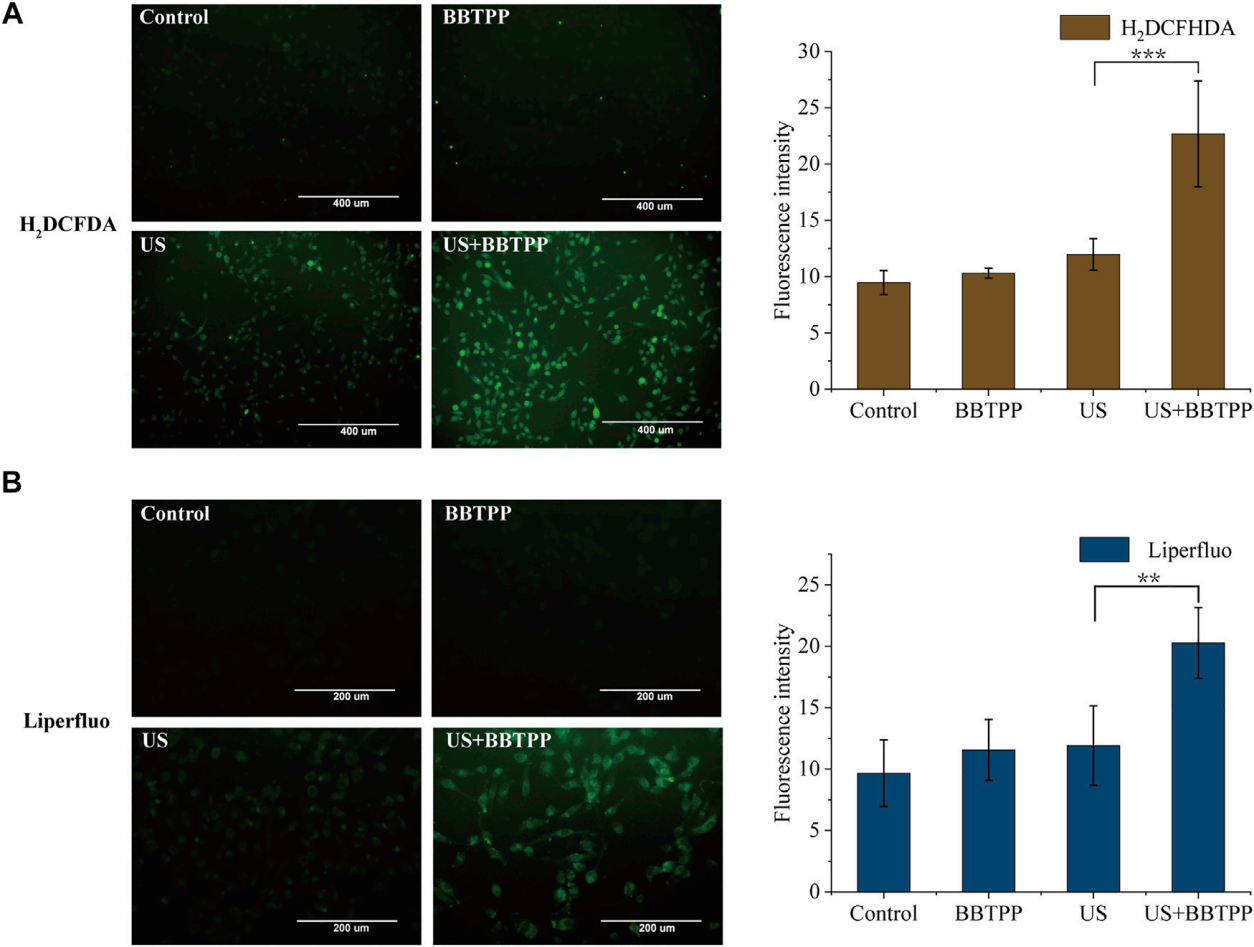
Intracellular ROS production and lipid peroxidation of PC-9 cells under different treatments **(A)** Representative images of intracellular ROS production measured by H_2_DCFDA and the fluorescence intensity of H_2_DCFDA analyzed by ImageJ **(B)** Representative images of lipid peroxidation measured by Liperfluo and the fluorescence intensity analyzed by ImageJ. The data were presented as the mean ± standard deviation. US: ultrasound; **p<0.01, ***p<0.001.

The intracellular generation of ROS was detected and quantified by the fluorescent probe H_2_DCFDA. As shown in Figure 3A, the SDT treatment resulted in an increase in H_2_DCFDA fluorescence intensity as compared to control, drug, and US samples. Cells in the sonication group also demonstrated slightly higher fluorescence, though much lower than that of SDT.

Lipid peroxidation was determined using the fluorescent sensor Liperfluo (Figure 3B). Using fluorescent microscopy, we found markedly enhanced fluorescence intensity of cells in the SDT group. A slight increase in the Liperfluo signal was also shown in ultrasonic cells. No obvious increase of fluorescence of control and BBTPP cells were observed.

### Apoptosis-Related Protein Expression

**FIGURE 4:**
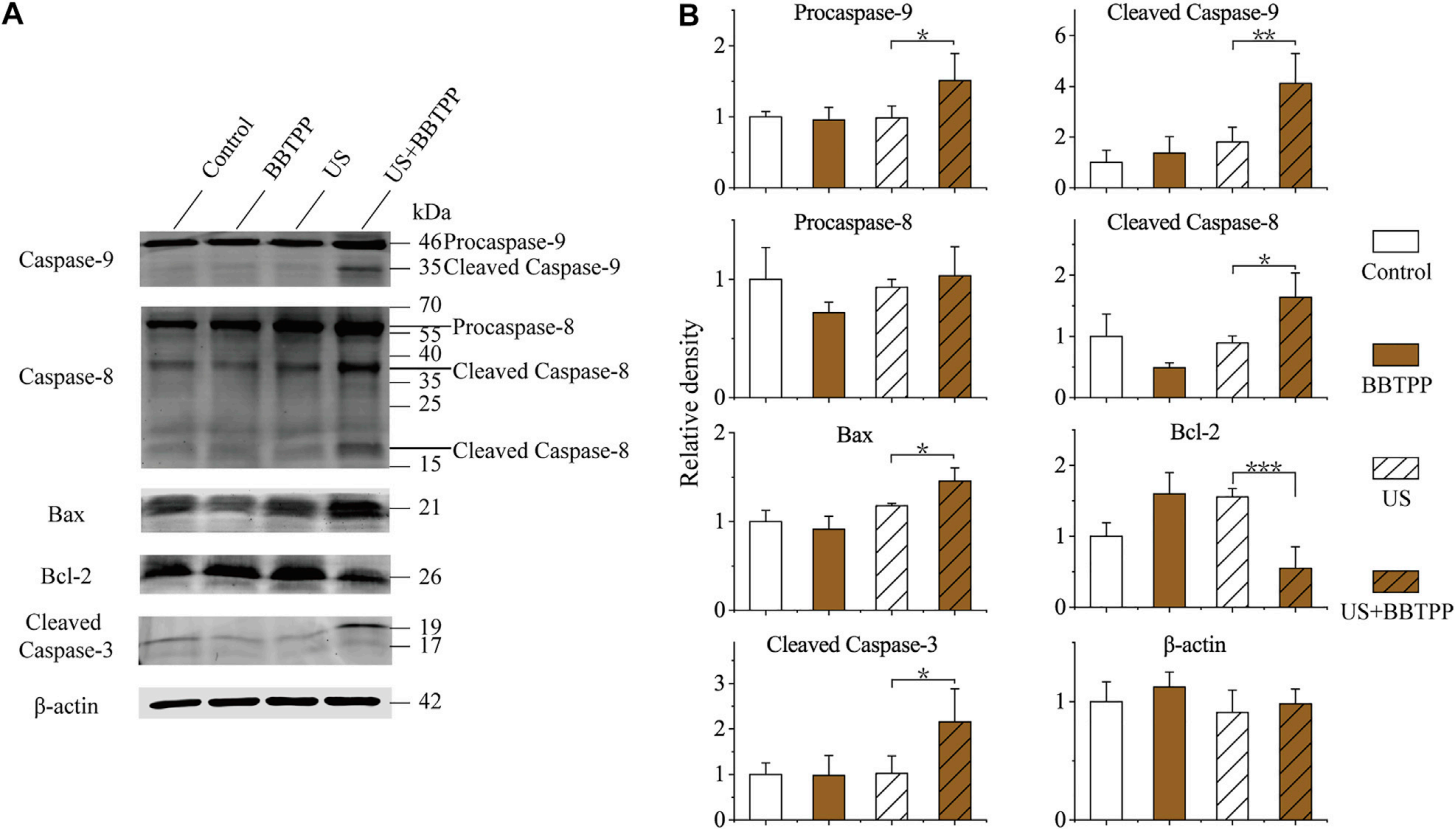
Western blot analysis of endogenic expression of caspase-9, caspase-8, Bax, Bcl-2, cleaved caspase-3, and *β*-actin in PC-9 cells with different treatments **(A)** Representative immunoblotting images **(B)** Relative expression of apoptosis-related proteins. The data were presented as the mean ± standard deviation. US: ultrasound; *p<0.05, **p<0.01, ***p<0.001.

The expression levels of apoptosis-related proteins (cleaved caspase-3, caspase-8, caspase-9, Bcl-2, and Bax) were examined in the control group, sonication group, and BBTPP with or without exposure to US exposure (Figure 4). The result showed strong expression levels of cleaved caspase-9, cleaved caspase-8, Bax, cleaved caspase-3 in the BBTPP-SDT sample when compared with the control, BBTPP, and sonication groups. Furthermore, the expression of Bcl-2 was suppressed by SDT. The expression of *β*-actin served as the internal loading control.

## Discussion

Our results showed that BBTPP could be effectively accumulated in PC-9 cells. In the presence of 4 μg/ml BBTPP, PLIFU provoked apparent apoptosis to PC-9 cells, while the cytotoxicity was not obvious when PLIFU was acting alone. HMME has been explored as a potent sonosensitizer in serval reports. The apoptosis rate of U937 cells under sonication (1.1 MHz, 1 W/cm^2^, continuous focused ultrasound) raised from 15.8 to 35.6% with the aid of HMME (Su et al., 2013). Less than 15% apoptotic rate in QBC939 cells induced by (1.2 MHz, 1 W/cm^2^, PLIU) in the presence of a wide dose range of HMME was also reported (Liang et al., 2013). Under our US apparatus, the sono-toxicity of BBTPP is significantly more effective than HMME. In our study, the cell death induced by SDT was mainly early apoptosis rather than late apoptosis and necrosis, which could be mainly attributed to that low-intensity ultrasound were more likely to cause early apoptosis (Guo et al., 2019; Zhang et al., 2021). The ratio of necrosis in cells increased with raising ultrasound intensity (Ferril et al., 2005). In addition, ultrasound in continuous mode or in tone burst mode with higher intensity or duty cycle was more prone to induce late apoptosis and necrosis (Su et al., 2013; Shen et al., 2020). Another study showed that PLIU related SDT mainly induced apoptosis, while high intensity induced necrosis (Lv et al., 2017). Besides ultrasonic apparatus, the time of incubation after treatment might also be responsible. Our apoptotic detection was immediately prepared after the completion of all treatments. Studies with 6 h incubation period after SDT showed a higher percentage of late apoptosis and necrosis (Sun et al., 2015a; Sun et al., 2015b).

We need to make clear that the overall apoptotic rate in our study had been greatly limited by the volume of the focal spot of the PLIFU system. The volume of the focal region is about 6.9 mm^3^ in our PLIFU apparatus. Thus, only a small percentage of cell suspensions could be irradiated. In fact, a substantial percentage of necrosis cells were observed in flow cytometry after we had employed a plane wave US to seek better coverage of US exposure (data not shown). In the meanwhile, the consistency of the results was drastically reduced as well. This situation might be attributed to the formation of standing waves which could cause more severe cavitation damage to tumor cells.

The only structural difference between BBTPP and HMME was that the BBTPP molecule contains a bigger number of ether groups. It is reasonable to deduce that the superior sono-toxicity of BBTPP is contributed by the active sites in the side chains. Ultrasonic cavitation is known to induce localized high-temperature environments-near cavitation cavities which are called “hotspots” (Suslick et al., 2002). At this moment, the cavity interface can be viewed as suitable chemical reactors for the oxidation of ether groups where they could be easily oxidized and then converted to peroxyl radicals. It is worth noting that the ether groups process much lower oxidative stability which led to several orders higher reaction rate than hydrocarbons at high-temperature conditions (Burke et al., 2015; Rodriguez et al., 2015). Porphyrin-based peroxyl radicals have long lifetimes compared to most other radical species and hence are able to induce oxidative damage to both surface and intracellular organelles (Misík and Riesz, 2000).

Possible participation of peroxyl radicals was also suggested as evidenced by a significant increase of both intracellular ROS and lipid peroxidation levels of BBTPP-SDT treated cells. ROS acts as a double-edged sword for cancer life: at moderated levels, ROS could promote cancer progression; at high levels, ROS could trigger programmed cell death of cancer cells (Perillo et al., 2020). Multiple pathways have been involved in ROS-induced cell death. Overproduction of ROS could cause direct damage to lipids, proteins, and nucleic acids, leading to cell death (Uttara et al., 2009; Liu et al., 2018). Various studies demonstrated that the excessive production of ROS in SDT plays an important part in killing cancer cells (Chen et al., 2014; Giuntini et al., 2018).

Lipid peroxidation of biological membranes can disturb the physical properties of lipid bilayers, which inevitably will alter lipid-lipid, and lipid-protein interactions, ion gradients, membrane fluidity and permeability, membrane-initiated signaling pathways (Gaschler and Stockwell, 2017). To gain further insight into the cytotoxic mechanism of BBTPP-SDT, specific signaling pathways involved in apoptosis were evaluated by western blot. There are two main apoptotic pathways: the extrinsic and intrinsic pathways (Elmore, 2007). Regulation of apoptosis is controlled by two classes of caspases: initiator caspases including caspase-8 and caspase-9, and effector caspases including caspase-3. Caspase-8 activation is related to the extrinsic apoptotic pathway, while caspase-9 is activated in the intrinsic pathway (Olsson and Zhivotovsky, 2011). Peroxidation of membrane lipids could cause cell apoptosis through both the extrinsic and intrinsic apoptotic pathways (Gaschler and Stockwell, 2017; Elkin et al., 2018). A classic feature of apoptotic cells is the dissipation of membrane lipid asymmetry and exposure of phosphatidylserine on the outer surface of the plasma membrane. The oxidized lipid could influence the membrane function (Volinsky and Kinnunen, 2013) and could depolarize mitochondria and disturb permeability by changing the expression of pro-apoptotic and anti-apoptotic proteins. Both Bcl-2 and Bax proteins belong to the Bcl-2 family, the former protein is anti-apoptotic and the latter one is pro-apoptotic pore former (Kale et al., 2017). In our study, the intrinsic pathway was activated through downing regulating Bcl-2 and releasing Bax, which increased mitochondrial membrane permeability ultimately causing the activation of caspase-9. The extrinsic pathway is activated through caspase-8. Both caspase-9 and caspase-8 then activate caspase-3, which executed apoptosis.

PDT, as a minimally invasive therapeutic modality, has been used for the management of a variety of diseases. However, this treatment could only apply to superficial lesions for the lacking penetration of light, which could be compensated by US. To improve the efficacy of SDT, optimizing US apparatus and sonosensitizers is equally important. For acquiring more effective sonosensitizers, in recent years, serval strategies associated with the photosensitivity of porphyrin macrocycles had been adopted (Qian et al., 2016; F et al., 2018; Pan et al., 2018). These strategies rely on the participation of sonoluminescence which only occurs in the most violent cavitation bubbles. It is well known that the structure and elastic properties of cells are widely different. Efforts even have been made to selectively damage tumor cells by means of the properties difference between tumor cells and normal cells (Mittelstein et al., 2020). Theories have been developed to describe the mechanism for bioeffects of therapeutic US (Zinin et al., 2005; Krasovitski et al., 2011). For instance, an intramembrane cavitation theory suggested that the cavitation cavities may be surrounded by elastic lipid membranes (Krasovitski et al., 2011). Meanwhile, there is little evidence to report the observation of intracellular sonoluminescence in the absence of artificial bubbles. We believe that this might be attributed to the viscoelasticity of the cellular components which greatly impairs the resonance strength of the intracellular cavities. Our *in vitro* data presented here indicate the importance of the side-chain structure of porphyrins on the US-induced ROS generation as well as the sono-toxicity of PC-9 cells. This method of improving sonosensitivity does not rely on sonoluminescence, so we believe that it is a promising approach to enhance the efficacy of low-intensity ultrasound-mediated SDT. There are limitations in our study. We only validate our approach *in vitro*. A systematic and comprehensive study containing *in vivo* evaluations can provide more detailed information. Furthermore, chemical analysis on the structure change of sonosensitizer in this study should be carried out in the future to further elucidate the reaction dynamics of the side chain.

## Conclusion

Our results indicate that BBTPP could accumulate in PC-9 cells after incubation. The sono-toxicity on PC-9 cells of BBTPP is more effective than HMME under the irradiation of PLIFU suggesting a pivotal role of the side-chain structure. Excessive intracellular ROS production and subsequent lipid peroxidation were induced by BBTPP-mediated SDT. The apoptosis of PC-9 was followed, and both extrinsic and intrinsic apoptotic pathways were involved. Our study indicates that, under PLIFU, BBTPP is valuable as a sonosensitizer in PC-9 cells.

## Data Availability

The original contributions presented in the study are included in the article/Supplementary Material further inquiries can be directed to the corresponding author.
