# Supplementary material for: Sonodynamic Effects of a Novel Ether-Group Modified Porphyrin Derivative Combined With Pulsed Low-Intensity Ultrasound on PC-9 Cells
Source: Front Pharmacol. 2021 Dec 6;12:792360. doi: 10.3389/fphar.2021.792360 (PMC8685451; doi:10.3389/fphar.2021.792360)
Supplement: Supplementary file 1 [file DataSheet1.PDF]

Supplementary Material

Supplementary Figures

Methods for detection of intracellular fluorescence:

1. Incubate PC-9 cells with different drugs for 2h
2. Wash cells with PBS two times
3. Trypsinized and resuspended into a quartz fluorescence cuvette
4. The fluorescence spectra under 402 nm excitation are detected by the fluorescence spectrophotometer (Varian Cary Eclipse)

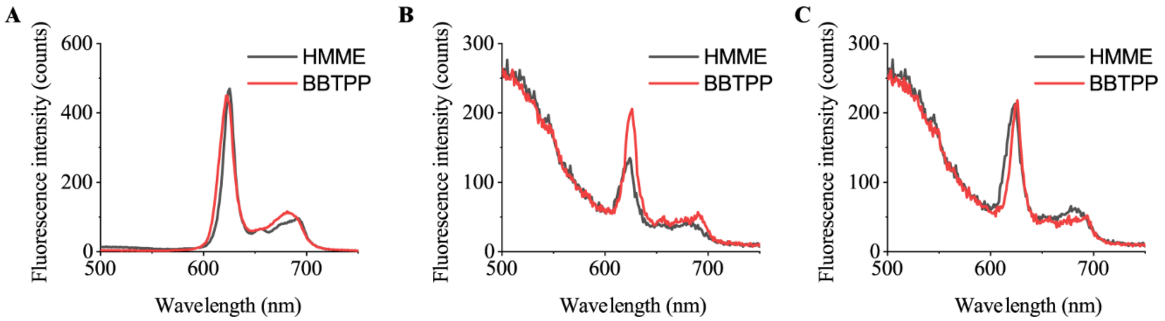

Supplementary Figure 1. This figure shows the fluorescence spectra under the 402 nm excitation for normalizing the cellular uptake of HMME and BBTPP. **(A)** Fluorescence spectra of HMME (4µg/mL) and BBTPP (4µg/mL) solutions. **(B)** Fluorescence spectra of PC-9 cell suspensions after incubation with culture medium containing 4µg/mL HMME and BBTPP. **(C)** Fluorescence spectra of PC-9 cell suspensions after incubation with culture medium containing HMME (8µg/mL) and BBTPP (4µg/mL).
